# The relationship between multisensory stimulus-integrated foreign language learning models and students’ psychological states and language skill development-an empirical analysis using the global learning assessment database

**DOI:** 10.3389/fpsyg.2025.1639885

**Published:** 2025-08-06

**Authors:** Liu Zhan, Jie Cheng

**Affiliations:** ^1^School of Health Management, Xianning Vocational Technical College, Xianning, China; ^2^School of Foreign Languages, Hubei University of Science and Technology, Xianning, China

**Keywords:** multisensory learning models, learning psychological states, foreign language learning, language skills, gender differences

## Abstract

**Introduction:**

This study explores the impact of multisensory stimulus-integrated foreign language learning models on students’ psychological states and the development of language skills. The research focuses on how different combinations of sensory stimuli—visual, auditory, and tactile—affect learning anxiety, motivation, self-confidence, and performance in listening, speaking, reading, and writing.

**Methods:**

An empirical analysis was conducted using data from the Global Learning Assessment Database. Quantitative methods, including regression analysis and Structural Equation Modeling (SEM), were employed to assess the effectiveness of multisensory learning models. The study examined the effects of specific sensory combinations and the integrated model on both psychological and linguistic outcomes, with additional analysis of gender-based differences.

**Results:**

Findings indicate that the visual—tactile model is particularly effective in reducing learning anxiety (regression coefficient = –0.52, *p* < 0.01), while the visual—auditory model significantly enhances learning motivation (regression coefficient = 0.48, *p* < 0.01). The fully integrated model (visual + auditory + tactile) yields the most balanced and significant improvements across all four language skills: listening comprehension (+32%, *p* < 0.001), speaking fluency (+33%, *p* < 0.001), reading comprehension (+31%, *p* < 0.001), and writing richness (+34%, *p* < 0.001). Gender analysis reveals that the integrated model most significantly boosts female motivation (coefficient = 0.51, *p* < 0.01) and male self-confidence (coefficient = 0.44, *p* < 0.01).

**Discussion:**

Multisensory learning models are shown to effectively enhance both psychological well-being and language proficiency. The integrated approach offers the most comprehensive benefits, providing strong empirical support and practical implications for optimizing foreign language instruction through sensory-rich strategies.

## 1 Introduction

In the context of accelerating globalization, foreign language learning has become one of the core competencies for enhancing individual international competitiveness. However, traditional foreign language learning models often rely on single sensory stimuli, such as auditory and visual input, while neglecting the impact of multisensory collaboration on learners’ cognitive and psychological states. Recent research in cognitive science and educational technology suggests that learning models integrating multisensory stimuli can effectively enhance learners’ immersion, improve the depth of language input processing, and promote the formation of long-term memory (Lo Buono et al., 2022; Shao et al., 2024). Therefore, exploring the mechanisms of multisensory stimuli in foreign language learning and their impact on students’ psychological states and language skill development is of significant theoretical and practical value. However, existing research is often conducted in a specific and single cultural or educational context, ignoring the fact that learners from diverse cultural backgrounds may have different preferences and responses to sensory input.

Existing research shows that multisensory learning models can activate different cognitive channels, allowing learners to acquire language in a more interactive and contextualized manner. For example, learning experiences that combine visual, tactile, auditory, and even olfactory and gustatory stimuli can not only enhance learning motivation but also positively influence psychological factors such as anxiety levels, self-confidence, and learning attitudes. Additionally, the effectiveness of foreign language learning is not only determined by the quantity of language input but is also constrained by the learner’s psychological state, such as anxiety levels, emotional engagement, and cognitive load (Wang et al., 2022). Therefore, investigating how multisensory stimuli affect learners’ psychological safety, self-efficacy, and emotional regulation abilities, thus promoting the development of language skills, is a critical area of current research.

To further validate the practical effectiveness of multisensory stimuli in foreign language learning, this work conducts an empirical analysis based on the Global Learning Assessment Database (GLAD) (Khasawneh et al., 2024). GLAD includes data from language learners across multiple countries and regions, covers various dimensions such as the learning process, psychological states, and language abilities, and provides a reliable data foundation for systematic research. This work employs quantitative analysis methods to explore the impact pathways of multisensory learning models on students’ psychological states and language skill development. It also attempts to reveal the applicability of this model across different cultural contexts. The findings of this work not only contribute to the refinement of foreign language learning theories but also provide data support for educational practices, driving innovation and optimization in foreign language teaching methods.

## 2 Recent work

### 2.1 Review of the current status of multisensory learning

The study of foreign language learning models has long attracted attention from multiple disciplines, including linguistics, education, and psychology. Traditional foreign language teaching models primarily rely on visual and auditory inputs for language acquisition. For example, Montero-Perez (2022) examined the impact of audiovisual input on foreign language learning, and mainly analyzed the role of screen text (subtitles) in language acquisition. They concluded that subtitles aided in vocabulary understanding and listening skills, but their effectiveness was influenced by learners’ cognitive load and language proficiency. This highlights the supportive role of audiovisual materials in traditional foreign language teaching. Zhang and Zou (2022) conducted a systematic review of multimedia input models and effectiveness in foreign language learning, covering multimodal resources such as text, audio, and video. Their research showed that multimedia technology could improve learners’ language comprehension abilities, but its effectiveness varied due to individual learner differences and teaching design. This emphasizes the necessity of technology assistance in traditional teaching models. Chen (2023) explored the relationship between learning styles and foreign language learning, and found that visual, auditory, and kinesthetic learners exhibited different adaptability in language learning. They suggested that traditional foreign language teaching should adapt teaching strategies to individual learning styles to enhance learning outcomes. This stresses the importance of personalized teaching. Lin (2024) reviewed the impact of learning styles on second language acquisition, and analyzed cognitive, motivational, and cultural factors. They argued that traditional foreign language teaching needed to focus on individual differences and adopt diverse teaching strategies to meet the needs of different learners. This provides a theoretical basis for optimizing foreign language teaching models.

In contrast, the multisensory learning model emphasizes promoting deep processing and long-term memory storage through various sensory channels, including touch, motion, and even smell. The core theoretical basis behind this lies in the “Dual-Coding Theory” and “Cognitive Load Theory” in cognitive science. The Dual-Coding Theory holds that processing information through both verbal (such as auditory) and non-verbal (such as visual) channels simultaneously can create richer mental representations, thereby enhancing memory. Furthermore, the synergistic effect of multisensory learning is not merely a simple superposition of multiple single-channel inputs but an interactive enhancement (Chit et al., 2024). This cross-channel integration is believed to reduce the cognitive load of a single channel and deepen understanding through complementary information. For example, in language learning, seeing an image of an object (visual), hearing its name (auditory), and touching its physical model (tactile) – these three inputs mutually confirm each other, forming a strong and stable memory network. The multisensory learning theory suggests that engaging multiple senses during the learning process can enhance memory retention and retrieval of information, and numerous scholars have conducted related research. For example, Aulina et al. (2024) explored the impact of Whole Brain Teaching and multisensory learning environments on cognitive development and examined the regulatory role of children’s learning preferences. They found that multisensory stimulation could promote knowledge, understanding, and memory, and improve motivation. This supports cognitive science-based teaching optimization strategies.

### 2.2 Current application of multisensory learning in foreign language education

In the field of foreign language learning, the multisensory learning model has been validated in various teaching practices. There are diverse ways to implement multisensory methods in classroom teaching. For example, Ahmad et al. (2024) examined the role of multisensory learning environments in promoting the learning skills of students with learning difficulties in the Asir region. The study found that teaching methods combining visual, auditory, and tactile stimuli significantly improved learning outcomes. Especially in foreign language learning, this teaching method has a positive impact on vocabulary memory and phonetic perception, which provides practical evidence for special education. Khasawneh (2024) also employed a case study method to explore the adaptability of multisensory technologies for English learners with dyslexia. The results indicated that teaching strategies combining tactile, auditory, and kinesthetic learning effectively improved reading skills and vocabulary mastery. This highlights the inclusiveness and adaptability of multisensory methods in foreign language education. Yan and Yan (2025) developed an English teaching model in a biomechanical environment and investigated how multisensory interaction could optimize language acquisition. Their research showed that learning approaches incorporating kinesthetic perception, auditory input, and visual feedback could enhance students’ ability in phonetic recognition and syntactic comprehension. This offers a novel interdisciplinary perspective for foreign language teaching.

### 2.3 Current research on psychological states in foreign language learning

Foreign language learning involves not only cognitive abilities but also emotional factors such as learning anxiety, motivation, and self-confidence. Many scholars have conducted research in this area. Hsu et al. (2024) explored the role of artificial intelligence (AI) image recognition and self-regulated learning strategies in English vocabulary acquisition. They focused on examining the impact of this approach on learning anxiety and learning behaviors. The findings suggested that AI-based autonomous learning strategies could effectively reduce learning anxiety, enhance learners’ vocabulary mastery ability, and optimize their learning behavior patterns. Teng (2024) analyzed the relationship between anxiety, self-efficacy, and learning motivation in online English learning environments. The results demonstrated that learners with high self-efficacy experienced lower anxiety levels and were more proactive in learning, while foreign language anxiety suppressed motivation and negatively affected learning outcomes. This emphasizes the critical role of psychological factors in online foreign language learning. Chan et al. (2025) investigated the impact of foreign language learning anxiety on Chinese students with English learning difficulties, particularly in terms of word reading ability. The study found that high anxiety levels weakened phoneme-grapheme mapping skills and affected reading accuracy and fluency. This emphasizes that reducing anxiety in foreign language teaching is crucial for improving reading ability and overall learning outcomes. Saidah (2024) examined the impact of academic self-confidence on English learners’ learning processes after the pandemic. The study concluded that students with higher self-confidence demonstrated greater autonomy in learning and were more engaged in classroom interactions. However, learners with low self-confidence were more likely to experience anxiety and avoidance tendencies, which could negatively impact the efficiency of language acquisition. Ardhana and Sadikin (2025) used role-playing activities to enhance the self-confidence of English-speaking learners. They found that immersive role-playing not only reduced speaking anxiety but also strengthened learners’ language expression skills in real-world communication contexts, thus promoting improvements in English speaking abilities.

### 2.4 Research gaps and innovations in this study

Through the analysis of the aforementioned literature, it is evident that existing multisensory learning research mainly focuses on the role of individual senses, such as the influence of subtitles on auditory comprehension or the promotion of learning outcomes through multimedia technologies. However, there is a lack of systematic analysis on how multisensory integration works synergistically in language acquisition. Additionally, existing studies tend to focus on specific groups, such as individuals with visual impairments or learning disabilities, with insufficient validation of the applicability of multisensory learning models for typical foreign language learners. Moreover, research on how multisensory learning affects foreign language learners’ psychological factors, such as anxiety, motivation, and self-confidence, remains fragmented and lacks a comprehensive theoretical framework for psychological regulation. In response to these gaps, the innovations of this work are reflected in three key aspects: First, this work utilizes large-scale survey data from the GLAD, combined with the theoretical framework of multisensory learning models, to quantitatively analyze the impact of multisensory learning on foreign language learners’ psychological states and learning outcomes. Second, the work expands the sensory scope researched by exploring the role of tactile, kinesthetic, and other sensory inputs in foreign language learning, and offers a more comprehensive analysis of the cognitive mechanisms involved. Finally, from an interdisciplinary perspective integrating psychology, education, and computer technology, this work constructs a model of the effects of multisensory learning on foreign language learners’ psychological states, and provides a scientific basis for the future optimization of teaching models.

## 3 Materials and methods

This work aims to explore the impact of a foreign language learning model that integrates multisensory stimuli on students’ psychological states and language skill development. To achieve this goal, it adopts a quantitative research approach. The work utilizes data from the GLAD to analyze the performance and psychological responses of foreign language learners from different countries and regions under a multisensory learning model.

### 3.1 Data sources and sample selection

The data used are sourced from the GLAD. GLAD is a comprehensive educational assessment platform that aggregates education data from various countries and regions, with a particular focus on performance in foreign language learning. The database includes student foreign language learning scores, psychological assessments (such as anxiety, self-confidence, and motivation), and various background information (such as cultural background, gender, and age). By utilizing data from the GLAD, this work can access a globally representative sample of foreign language learners, covering students from different regions, cultures, and language backgrounds, making it highly representative and generalizable.

The sample selection for this work targets university students, with an expected sample size of 1,030 participants. To ensure the representativeness of the data, the samples are selected from the GLAD according to the inclusion and exclusion criteria shown in Table 1. This ensures a balance of variables such as region, cultural background, gender, and age. For example, students from Europe, North America, Asia, and Africa are chosen to compare the effects of multisensory learning models across different cultural contexts. Additionally, to ensure sample heterogeneity, the work also considers students’ language proficiency and selects a multi-level sample that includes both beginner and advanced foreign language learners.

**TABLE 1:** Inclusion and exclusion criteria for the sample in the database.

Standard category	Inclusion criteria	Exclusion criteria
Region	University students from Europe, Asia, Africa, and other regions.	Participants from regions not listed above.
Cultural background	University students from diverse cultural backgrounds, ensuring inclusion of East Asian, European, African, and other cultural groups.	Students from a single cultural background only.
Gender	Balanced gender ratio, with representation of both male and female students.	Severe gender imbalance (such as fewer than 10% of participants from one gender).
Age	University students aged between 18 and 25.	Participants outside the age range (such as under 18 or over 25).
Language proficiency	Includes both beginner and advanced foreign language learners, ensuring diversity in language proficiency levels.	Foreign language learners without basic language ability (such as being unable to engage in simple daily conversation).
Academic status	Current university students who have completed at least one semester of foreign language courses.	Non-university students or those who have not taken foreign language courses.
Data integrity	Complete data with no significant missing information; participants have filled out all necessary assessment scales and test items.	Severe data missing (such as inability to provide complete language skill ratings or psychological state scale responses).

During the sample selection from the database, anonymization is applied to the data of each participant to ensure confidentiality and compliance with data protection regulations. Additionally, all participants have signed informed consent forms prior to the use of their data, and their personal information will only be used for academic analysis of this study, not for any commercial purposes. Furthermore, the work addresses any missing data by performing appropriate imputation and data cleaning procedures to ensure the integrity of the data and the accuracy of the analysis.

### 3.2 Research design

This work adopts a quantitative research approach to comprehensively explore the impact of a foreign language learning model that integrates multisensory stimuli on students’ psychological states and language skills. The goal is to examine the mechanisms and practical effects of multisensory learning from multiple perspectives.

This work mainly adopts quantitative analysis methods, including regression analysis and structural equation modelling (SEM), to explore in depth the impact of multisensory learning models on students’ psychological states and language skills. Regression analysis initially tests the direct impact of different learning models as independent variables on various dependent variables (psychological states, language skills). SEM is used to test a more complex theoretical model, which systematically reveals the potential, multi-level causal path relationships between these variables.

Regression analysis is a statistical method used to study the relationship between independent variables (such as multisensory learning models) and dependent variables (such as students’ psychological states and language skills). Regression analysis helps quantify and test the strength and direction of the influence of different types of multisensory learning models on students’ psychological states and language skills. Here, the regression model is specified as follows in Equation (1):


(1) $$Y_{i}=\beta_{0}+\beta_{1}\,X_{1}+\beta_{2}\,X_{2}+\cdots +\beta_{k}\,X_{k}+\varepsilon_{i}$$

*Y*_*i*_ represents the dependent variable, which refers to students’ psychological states or language skills (such as anxiety, motivation, listening, and speaking). *X*_1_,*X*_2_,⋯,*X*_*k*_ represent the independent variables, which correspond to different types of multisensory learning models (such as visual + auditory, visual + tactile, and auditory + olfactory). β_0_ is the constant term, representing the baseline value when all independent variables are zero. β_1_,β_2_,⋯,β_*k*_ represent the regression coefficients, are the degree of influence each learning model has on the dependent variable. ε_*i*_ represents the error term, which accounts for the portion of the variance that the model does not explain.

SEM is a multivariate statistical technique that integrates factor analysis and path analysis. It can handle both directly measurable observed variables (such as questionnaire scores and test results) and unobservable latent variables (such as psychological states and language skills). This makes it very suitable for verifying complex models based on theoretical assumptions. The model structure of SEM is represented as follows in Equation (2):


(2) Y=B⋅X+E

*Y* represents the dependent variable vector, that is, the latent variables, which include psychological states (such as anxiety and self-confidence) and language skills (such as listening and speaking). *X* refers to the independent variable vector, that is, the observed variables, representing different types of multisensory learning models (such as visual + auditory and visual + tactile). *B* is the path coefficient matrix, indicating the extent of the influence of the independent variables on the dependent variables. *E* represents the error term, accounting for the portion of the variance that the model does not explain.

The SEM constructed in this work aims to test the following core hypothesis. Multisensory learning models directly affect the development of language skills while indirectly influencing language skills through the mediating variable of students’ psychological states (learning anxiety, motivation, and self-confidence). Specifically, the model includes three core latent variables:

Exogenous latent variable: Multisensory learning models, whose observed indicators are the participation frequency and experience scores reported by students under different learning models (such as “visual + auditory”, “visual + tactile”, etc.).

Mediating latent variable: Psychological states, whose observed indicators are students’ scores on the “Foreign Language Learning Anxiety Scale”, “Learning Motivation Scale”, and “Language Self-Confidence Scale”. These three indicators are selected because existing literature has repeatedly confirmed that learning anxiety, motivation, and self-confidence are key psychological factors affecting foreign language learning outcomes.

Endogenous latent variable: Language skill development, whose observed indicators are students’ standardized test scores in the four dimensions of listening, speaking, reading, and writing.

The construction and evaluation of the model follow a standard process: first, hypothetical paths between variables are set based on theoretical literature; then, the maximum likelihood method is used to estimate model parameters; finally, the degree of matching between the model and the data is evaluated through a series of goodness-of-fit indices. They include chi-square/degree of freedom ratio (χ^2^/df < 3), comparative fit index (CFI > 0.90), Tucker-Lewis index (TLI > 0.90), root mean square error of approximation (RMSEA < 0.08), and standardized root mean square residual (SRMR < 0.08). These steps ensure the scientific testing of the complex mechanism through which multisensory learning models affect language skills through psychological states.

### 3.3 Variable definition and measurement

The definition and measurement of variables are crucial as they directly affect the results of data analysis and the validity of the research. To accurately assess the impact of multisensory learning models on students’ psychological states and language skill development, it is first necessary to clearly define and determine the measurement methods of the independent, dependent, and control variables.

When defining and measuring the variables in this work, special attention is paid to the operationalization of latent variables. Based on the analysis of the current research status of psychological states, learning anxiety, learning motivation, and self-confidence are generally regarded as the three core psychological constructs that affect foreign language learners’ engagement, persistence, and final achievements. For example, studies have shown that high anxiety inhibits learning effects, high motivation is the driving force for continuous learning, and self-confidence is directly related to learners’ performance in communicative tasks. Therefore, this work uses these three factors as observed indicators to measure the core latent variable of “psychological states.” Validated standardized scales (as exhibited in Table 2) are adopted to ensure the reliability and validity of the measurement, thereby providing a reliable data foundation for the construction of SEM.

**TABLE 2:** Variable measurement.

Variable category	Variable name	Measurement tool	Measurement method
Independent variables	Visual + auditory	Self-report questionnaire	Likert five-point scale (1-5)
	Visual + tactile	Self-report questionnaire	Likert five-point scale (1-5)
	Auditory + olfactory	Self-report questionnaire	Likert five-point scale (1-5)
	Integrated mode	Self-report questionnaire	Likert five-point scale (1-5)
Dependent variables	Learning anxiety	Foreign language learning anxiety scale	Scale rating (1-5, intensity of anxiety)
	Learning motivation	Learning motivation scale	Scale rating (1-5, intensity of motivation)
	Self-confidence	Language self-confidence scale	Scale rating (1-5, level of confidence)
	Listening ability	Standardized listening test	Test score
	Speaking ability	Standardized speaking test	Test score
	Reading ability	Standardized reading test	Test score
	Writing ability	Standardized writing test	Test score
Control variables	Age	Self-report	Age (in years)
	Native language background	Self-report	Native language category (grouped by language)
	Study duration	Self-report	Weekly study hours (hours)

By precisely defining and measuring the independent variables, dependent variables, and control variables, this work ensures that the effects of the multisensory learning model are evaluated scientifically and objectively. The independent variables are measured through self-report questionnaires, while the dependent variables are assessed using standardized tests and scales. The control variables help mitigate the impact of external confounding factors.

### 3.4 Data statistical analysis

This work explores the impact of multisensory learning models on students’ psychological states and language skills through regression analysis and SEM. Analysis of Variance (ANOVA) and regression analysis are used to test the effects of different learning models on students’ psychological states and language skills. ANOVA can assess the differences in how various types of sensory stimuli affect students’ skills (such as listening, speaking, reading, and writing). Regression analysis is used to examine the relationship between different multisensory learning models (such as visual + auditory and visual + tactile) and the dependent variables (psychological states and language skills). By constructing regression models, the specific impacts of each learning model on students’ psychological and language abilities are quantified.

In addition, to further explore the potential causal relationships between the variables, this work also employs SEM. SEM is capable of handling the complex causal relationships between multiple variables and modeling latent variables. In this model, the model simultaneously considers both the direct and indirect effects of multisensory learning models, thus providing a more comprehensive assessment of their multi-layered impact on students’ learning outcomes. Through these analytical methods, the work can provide strong evidence for the effectiveness of multisensory learning models. In all statistical tests of this work, the statistical significance level (alpha level) is set at α = 0.05. Therefore, when the *p*-value is less than 0.05, the research results can be considered statistically significant, and the null hypothesis can be rejected.

## 4 Results and discussion

### 4.1 Analysis of the impact of learning models on students’ psychological states

**FIGURE 1:**
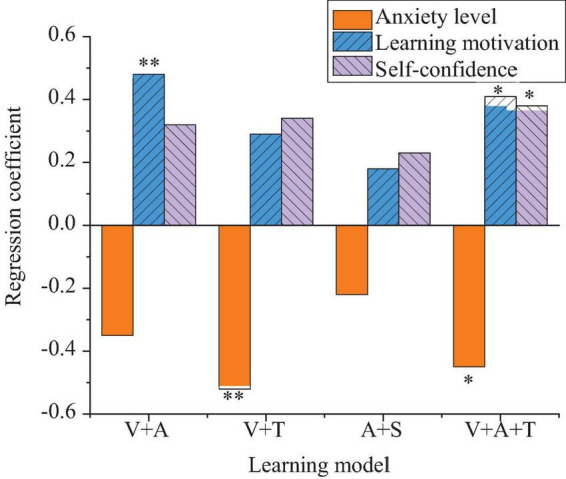
Results of students’ psychological states under multisensory learning models (**p < 0.01, *p < 0.05).

This section analyzes the impact of different types of multisensory learning models on students’ psychological states, including anxiety, motivation, and self-confidence. Figure 1 presents the detailed results.

In Figure 1, the results of the regression analysis show that the learning model combining visual and tactile senses is particularly effective in reducing learning anxiety, with a negative regression coefficient of −0.52 (*p* < 0.01). This indicates that tactile sensory perception and learning feedback help alleviate students’ foreign language learning pressure. For learning motivation, the visual + auditory model stands out, with a regression coefficient of 0.48 (*p* < 0.01). This model stimulates students’ motivation by enhancing their sense of participation and focus. The integrated model (visual + auditory + tactile) shows a balanced effect in reducing anxiety and boosting self-confidence, contributing to emotional regulation and learning engagement (*p* < 0.05). Therefore, the analysis of variance further confirms that there are significant differences in the impact of different sensory combinations on psychological states. The visual + tactile combination model is particularly effective in reducing anxiety.

### 4.2 Analysis of the impact of multisensory learning models on language skills

**FIGURE 2:**
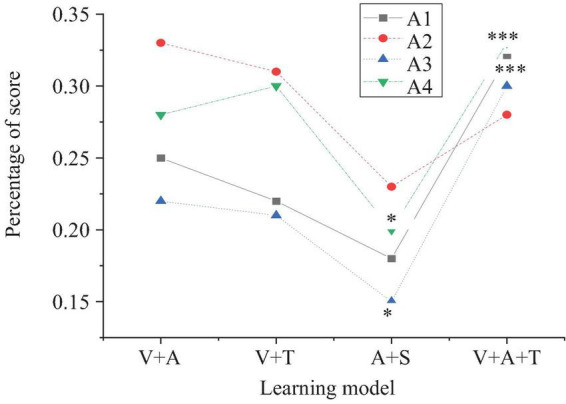
The results chart of the impact of multisensory learning model on students’ foreign language listening skills (A1: improvement in listening comprehension scores; A2: improvement in listening memory retention; A3: improvement in listening response speed; A4: improvement in audio recognition) (*p < 0.05, ***p < 0.001).

**FIGURE 3:**
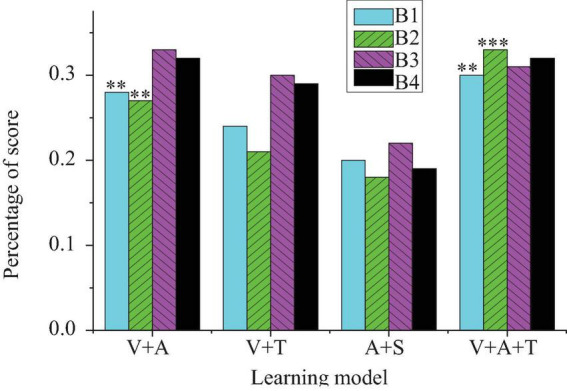
The results chart of the impact of multisensory learning model on students’ foreign language speaking skills (B1: improvement in pronunciation accuracy; B2: improvement in speaking fluency; B3: improvement in grammatical accuracy; B4: improvement in vocabulary usage richness) (**p < 0.01, ***p < 0.001).

**FIGURE 4:**
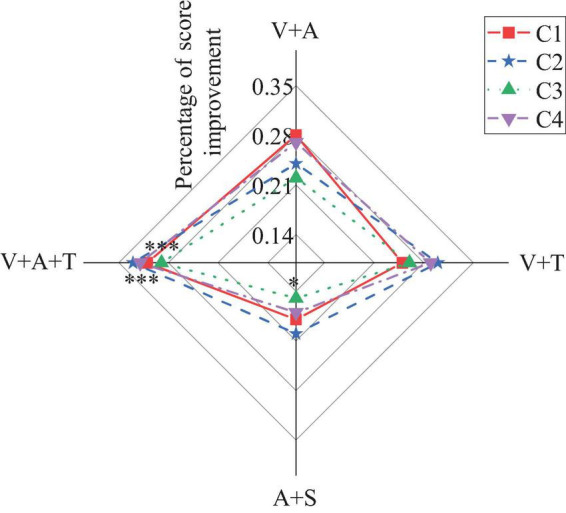
The results chart of the impact of multisensory learning model on students’ foreign language reading skills (C1: improvement in reading comprehension scores; C2: improvement in mastery of grammatical structures; C3: improvement in vocabulary mastery; C4: enhancement of information extraction and summarization skills) (*p < 0.05, ***p < 0.001).

**FIGURE 5:**
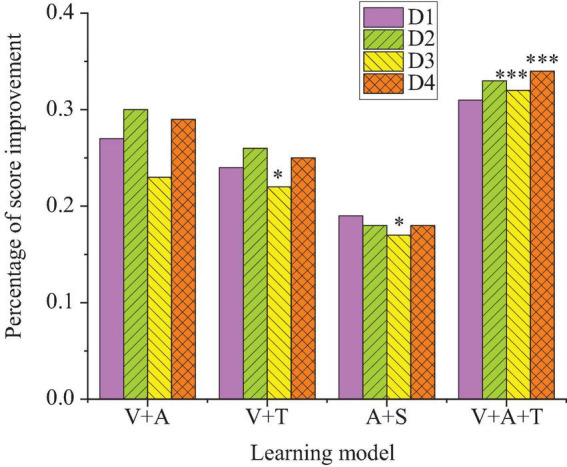
The results chart of the impact of multisensory learning model on students’ foreign language writing skills (D1: improvement in clarity of writing expression; D2: improvement in writing structure and organization; D3: enhancement of creative thinking; D4: improvement in the richness of writing content) (*p < 0.05, ***p < 0.001).

This section further explores the effects of multisensory learning models on students’ foreign language learning skills, including listening, speaking, reading, and writing. Figures 2–5 present the detailed results.

In Figure 2, the integrated model (visual + auditory + tactile) demonstrates the most significant improvement in listening skills, especially in listening comprehension scores (32%, *p* < 0.001) and audio recognition (33%, *p* < 0.001). This improvement may be attributed to the multiple sensory stimuli from vision, hearing, and touch, which enhance students’ perception and response abilities. In contrast, the combined auditory and olfactory model shows relatively weaker improvements, particularly in listening response speed (15%, *p* < 0.05) and audio recognition (20%, *p* < 0.05), with only limited enhancement in these areas.

In Figure 3, regarding oral skills, the combination of visual and auditory models performs better in improving pronunciation accuracy (28%, *p* < 0.01) and speaking fluency (27%, *p* < 0.01). The integrated model, however, shows a more balanced performance, with relatively even improvements across all oral skill dimensions [pronunciation accuracy increases by 30%, and speaking fluency increases by 33% (*p* < 0.001)]. In comparison, the visual + tactile model shows slightly less improvement in oral skills, especially in terms of vocabulary richness (29%) and grammatical correctness (30%, *p* < 0.01). Although there is some effect, it is still not as significant as the integrated mode.

Figure 4 reveals that in terms of reading skill improvement, the integrated model also performs exceptionally well, especially in enhancing reading comprehension scores (31%, *p* < 0.001) and the ability to extract and summarize information (32%, *p* < 0.001), surpassing other combination models. The single auditory + olfactory combination model performs relatively weakly across all dimensions, particularly in the improvement of vocabulary mastery (15%, *p* < 0.05), where the effect is the most limited.

In Figure 5, concerning writing skills, the integrated model (visual + auditory + tactile) also leads, with improvements in all dimensions exceeding 30%, especially in the enhancement of writing content richness (34%, *p* < 0.001) and the development of creative thinking (32%, *p* < 0.001). This demonstrates the comprehensive effect of this mode. In comparison, the visual + tactile and auditory + olfactory combinations show relatively lower improvement effects, particularly in the development of creative thinking (22 and 17%, *p* < 0.05), where the gains are more limited.

Overall, the work clearly shows that different learning models have distinct effects on the improvement of language skills. The integrated model, through multisensory stimulation, provides a more balanced and significant effect across all language skills. Especially in the four dimensions of listening, speaking, reading, and writing, its performance surpasses that of other single-sensory or fewer-sensory combination models.

### 4.3 Analysis of the impact of multisensory learning models on language skills

**FIGURE 6:**
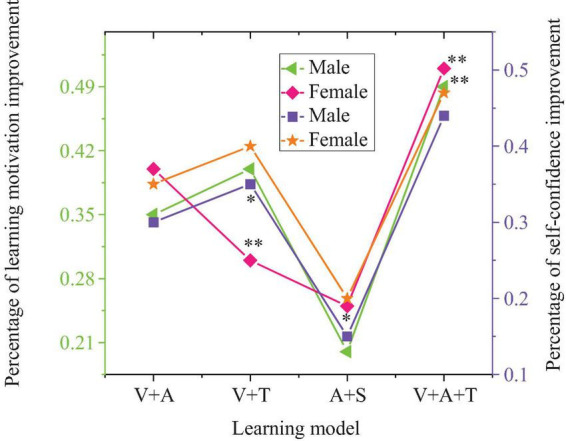
The impact of gender differences on learning motivation and self-confidence (*p < 0.05, **p < 0.01).

**FIGURE 7:**
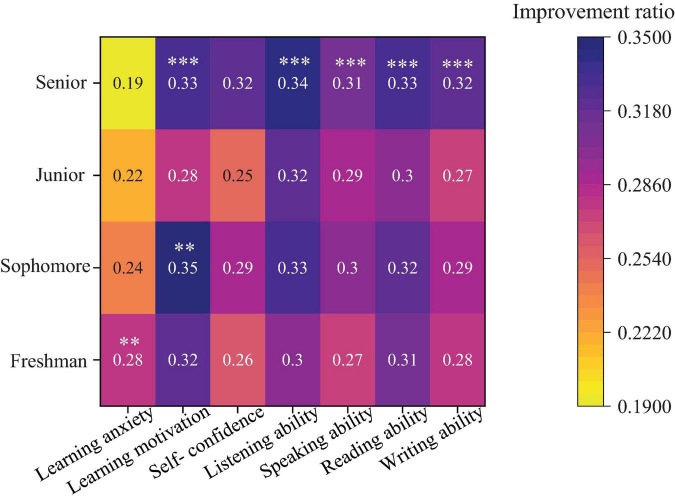
The relationship between age group, language skills, and psychological states (***p < 0.001, **p < 0.01).

Further analysis investigates the impact of gender differences on learning motivation and self-confidence. Figure 6 presents the specific results. Figure 7 shows the relationship between different age groups and language skills, and psychological states.

In Figure 6, there are certain differences in how learning models affect the learning motivation and self-confidence of males and females. The integrated model (visual + auditory + tactile) performs the best across all dimensions, with both male and female participants showing significant improvements in learning motivation and self-confidence. Notably, the improvement in learning motivation is most pronounced for females (0.51, *p* < 0.01), while males show the strongest improvement in self-confidence (0.44, *p* < 0.01). In contrast, the visual + tactile model provides a more balanced improvement in learning motivation and self-confidence for both males and females, particularly in boosting male self-confidence (0.35, *p* < 0.05) and female learning motivation (0.4, *p* < 0.01). The auditory + olfactory combination model is relatively weaker, with limited effects, particularly in improving male self-confidence (0.15, *p* = 0.120) and female learning motivation (0.25, *p* < 0.05). This suggests that single sensory stimulation has a weaker impact on regulating emotions and motivation. Overall, multisensory input models, especially the integrated model, have a stronger advantage in promoting learning motivation and self-confidence, and gender differences have a minimal impact on the effectiveness.

In Figure 7, with the increase of grades, students’ learning anxiety shows a statistically significant downward trend. The learning anxiety level of freshmen is the highest (28%, *p* < 0.01 compared with the graduating grade), while in the senior year, the anxiety level decreases significantly to 19%. Learning motivation reaches its peak in the sophomore year (35%, *p* < 0.01) and then remains at a high level. More importantly, students’ four language skills (listening, speaking, reading, and writing) show a steady and remarkable improvement with the increase of grades. Especially in the senior year, the improvement in all skill dimensions exceeds 32%, and compared with freshmen, these improvements reach extremely high statistical significance (*p* < 0.001). This illustrates that with the advancement of the academic process, students’ emotions and abilities tend to be stable and mature.

### 4.4 Discussion

This work’s analysis shows that interventions based on multisensory learning models have a significant effect on both students’ psychological states and language skills. In terms of psychological state, the combined visual and tactile learning model, in particular, has a marked advantage in alleviating students’ learning anxiety. The negative regression coefficient indicates that tactile stimulation can provide specific sensory feedback, effectively reducing anxiety in foreign language learning. This finding is consistent with existing research, such as Kidd et al. (2023), which states that tactile involvement helps students better regulate their emotions and lower anxiety levels. Furthermore, the visual and auditory combined model significantly enhances learning motivation by improving learning immersion and focus. The integrated model (visual + auditory + tactile) demonstrates more balanced effects. It helps to reduce anxiety and boosts confidence, promoting greater student engagement in learning. These results support the viewpoint of Song et al. (2024), who argue that multisensory stimulation not only alleviates anxiety but also effectively enhances learners’ emotional state and confidence, thereby improving learning outcomes.

In terms of language skills, the integrated model shows the most significant effects, particularly in the areas of listening, speaking, reading, and writing, all of which exhibit remarkable improvements. The integrated model not only provides balanced improvements across various dimensions of language skills, but also leads to greater progress in speaking fluency, reading comprehension, and information extraction compared to other sensory combinations. For example, in speaking, all indicators in the integrated model show an improvement of over 30%, especially in terms of creative thinking and the richness of writing content. This result aligns with the research result of Sanfilippo et al. (2022), which suggests that multisensory learning can enhance the depth and breadth of learning by providing rich sensory input. Moreover, an analysis of gender differences shows that although there are some differences between males and females in terms of improvements in learning motivation and confidence, the integrated model performs well in enhancing both genders’ motivation and confidence. Besides, females show more significant improvement in learning motivation. In summary, multisensory learning models, especially the integrated model, show balanced and significant improving effects across various language skills. Furthermore, these models effectively regulate students’ emotions and psychological states, encouraging them to engage more actively in language learning.

Although this work is based on the GLAD and strives for sample diversity, it must be acknowledged that the generalizability of the research results should be viewed with caution. Students from different cultural and educational systems may have significant differences in learning habits and sensory preferences. For instance, in collectivist cultures that rely heavily on context, learning models combining visual contexts and auditory inputs may be more popular; in individualistic cultures that focus on individual analysis, tactile models providing specific operational feedback may be more effective. The overall analysis of this work shows the universal superiority of the integrated model. However, it does not conduct in-depth comparative analysis on specific subgroups (such as those divided by country or cultural background). This is mainly because the current data sample size in some subgroups is insufficient to support robust statistical comparisons. Consequently, the findings of this work should be regarded as preliminary evidence of a general trend rather than a one-size-fits-all conclusion. Future research should conduct more targeted comparative experiments in different cultural contexts to reveal how cultural factors regulate the effects of multisensory learning.

From the perspective of cognitive mechanisms, this balancing effect may precisely reflect the synergy of multiple senses. Shams and Seitz (2008) stated that visual, auditory, and tactile inputs did not work in isolation but interacted in the brain to form stronger and more stable neural representations than single-sensory inputs. The results of this work echo the cross-modal integration theory proposed by Shams and Seitz (2008). For example, in the improvement of speaking fluency, the integrated model is significantly effective probably because learners not only hear correct pronunciations (auditory) and see mouth shapes and contexts (visual); they also perceive the rhythm of syllables through tactile devices (such as vibration feedback). This multiple confirmation greatly reduces cognitive load, making language output more automatic and fluent. Similarly, in the improvement of the richness of writing content, multisensory experiences provide students with more vivid and multidimensional materials, stimulating their association and creativity. Thus, the findings of this work provide empirical support from the field of foreign language education for the synergistic effect hypothesis of multisensory learning.

Regarding the analytical results of this work (especially SEM), which reveal significant structural relationships between multisensory learning models, psychological states, and language skills, the causal implications of these results must be interpreted with caution. This work adopts a quantitative analysis method based on cross-sectional survey data. Regression analysis and SEM essentially reveal correlational relationships between variables rather than strict causal relationships. For instance, the “visual + tactile” model is found to be associated with lower learning anxiety. However, this cannot completely rule out the possibility of reverse causality (i.e., students with lower anxiety levels are more inclined to choose or adapt to this interactive mode). It also cannot exclude the existence of unmeasured confounding variables (such as teachers’ teaching styles, classroom atmosphere, etc.) that simultaneously affect the choice of learning models and students’ psychological states. Hence, this work provides strong empirical correlational evidence for the theory that multisensory learning can improve psychological states and language skills. However, establishing its direct causal effects requires more rigorous research designs for verification.

## 5 Conclusion

This work investigates the impact of multisensory learning models on university students’ psychological states and language skills during foreign language learning. The results indicate that integrated multisensory stimuli (visual, auditory, and tactile) can significantly improve students’ learning anxiety, motivation, and confidence. Moreover, it can also lead to balanced and significant improvements in language skills such as listening, speaking, reading, and writing. Notably, the effects are particularly prominent in speaking fluency and writing content richness. Through an analysis of gender and grade-level differences, this work also reveals subtle differences in the effects of the multisensory learning model based on gender and grade level and further validates the different impacts of various sensory combinations on emotional regulation and learning outcomes. However, this work has some limitations. Firstly, and most importantly, this work is based on cross-sectional data. Although its analytical methods (regression analysis and SEM) can effectively test the relationship models between variables, they essentially establish correlations and cannot fully confirm causal relationships. This limitation means that the observed path relationships (such as multisensory learning models improving psychological states) need to be verified through more rigorous research designs. To address this limitation and validate the findings of this work, future research urgently needs to adopt an experimental research design. For example, researchers can recruit learners and randomly assign them to different experimental groups (e.g., the integrated multisensory model teaching group) and control groups (e.g., the traditional teaching model group). By conducting longitudinal follow-up measurements of the students’ psychological states and language skills in both groups before and after the intervention, potential confounding variables can be more effectively controlled. This allows for more reliable inference of the causal effects of multisensory teaching interventions. Secondly, as mentioned earlier, the sample of this work is limited to college students in the GLAD, which restricts the research results’ generalizability to a broader student population. Future research can expand the sample scope and incorporate key variables not covered by this work. These variables include: (1) the genetic relationship between the mother tongue and the target language; (2) learners’ cognitive styles and language intelligence levels; (3) learners’ readiness and types of learning motivation. By constructing a more complex moderated mediation model, it is possible to answer the more practically instructive question. “What combination of multisensory models produces the best effects through what psychological mechanisms for learners with what characteristics in what learning contexts?” It also explores the applicability and long-term effects of multisensory learning models in different disciplines and educational stages.

## Data Availability

The original contributions presented in this study are included in this article/supplementary material, further inquiries can be directed to the corresponding author.
